# Grandparents Raising Grandchildren in Appalachia: An Examination of Available Services

**DOI:** 10.1093/geroni/igaa057.2058

**Published:** 2020-12-16

**Authors:** Bradford Stucki

**Affiliations:** Virginia Tech, Blacksburg, Virginia, United States

## Abstract

Previous research indicates that higher numbers of grandparents raising grandchildren live in Appalachia, relative to the rest of the United States. These grandparents may have diverse needs that could benefit from services. When grandparents cannot access needed services, their well-being can be negatively affected. Using the “2017 GrandFacts: State Fact Sheets for Grandfamilies” for the 13 states defined as being part of Appalachia by the Appalachian Regional Commission, this study examined the types and availability of local services by Appalachian sub-region. Excluding state and federal public benefits, most common service types were emotional support, information and referral, financial assistance, and education. Least common service types included grandchild special health needs, legal services, and early childhood intervention. For service availability, four of the five Appalachian sub-regions had no services in over 65% of their counties. South and North Central Appalachia regions had no services in over 90% of their counties. Part of a symposium sponsored by the Grandparents as Caregivers Interest Group.

